# Alternative measures of trait–niche relationships: A test on dispersal traits in saproxylic beetles

**DOI:** 10.1002/ece3.10588

**Published:** 2023-10-19

**Authors:** Ryan C. Burner, Jörg G. Stephan, Lukas Drag, Mária Potterf, Tone Birkemoe, Juha Siitonen, Jörg Müller, Otso Ovaskainen, Anne Sverdrup‐Thygeson, Tord Snäll

**Affiliations:** ^1^ U.S. Geological Survey Upper Midwest Environmental Sciences Center La Crosse Wisconsin USA; ^2^ Faculty of Environmental Sciences and Natural Resource Management Norwegian University of Life Sciences Ås Norway; ^3^ SLU Swedish Species Information Centre Swedish University of Agricultural Sciences Uppsala Sweden; ^4^ Field Station Fabrikschleichach, Department of Animal Ecology and Tropical Biology, Biocenter University of Würzburg Rauhenebrach Germany; ^5^ Institute of Entomology Biology Centre of the Czech Academy of Sciences Ceske Budejovice Czech Republic; ^6^ Department of Life Science Systems Technical University of Munich Freising Bavaria Germany; ^7^ Natural Resources Institute Finland (Luke) Helsinki Finland; ^8^ Bavarian Forest National Park Grafenau Germany; ^9^ Department of Biological and Environmental Science University of Jyväskylä Jyväskylä Finland; ^10^ Organismal and Evolutionary Biology Research Programme, Faculty of Biological and Environmental Sciences University of Helsinki Helsinki Finland; ^11^ Department of Biology, Centre for Biodiversity Dynamics Norwegian University of Science and Technology Trondheim Norway

**Keywords:** Bayesian joint species distribution model, community‐weighted mean trait values, deadwood, dispersal capacity, morphological traits, phylogeny, response trait, wing length

## Abstract

Functional trait approaches are common in ecology, but a lack of clear hypotheses on how traits relate to environmental gradients (i.e., trait–niche relationships) often makes uncovering mechanisms difficult. Furthermore, measures of community functional structure differ in their implications, yet inferences are seldom compared among metrics. Community‐weighted mean trait values (CWMs), a common measure, are largely driven by the most common species and thus do not reflect community‐wide trait–niche relationships per se. Alternatively, trait–niche relationships can be estimated across a larger group of species using hierarchical joint species distribution models (JSDMs), quantified by a parameter Γ. We investigated how inferences about trait–niche relationships are affected by the choice of metric. Using deadwood‐dependent (saproxylic) beetles in fragmented Finnish forests, we followed a protocol for investigating trait–niche relationships by (1) identifying environmental filters (climate, forest age, and deadwood volume), (2) relating these to an ecological function (dispersal ability), and (3) identifying traits related to this function (wing morphology). We tested 18 hypothesized dispersal relationships using both CWM and Γ estimates across these environmental gradients. CWMs were more likely than Γ to show support for trait–niche relationships. Up to 13% of species' realized niches were explained by dispersal traits, but the directions of effects were consistent with fewer than 11%–39% of our 18 trait–niche hypotheses (depending on the metric used). This highlights the difficulty in connecting morphological traits and ecological functions in insects, despite the clear conceptual link between landscape connectivity and flight‐related traits. Caution is thus warranted in hypothesis development, particularly where apparent trait–function links are less clear. Inferences differ when CWMs versus Γ estimates are used, necessitating the choice of a metric that reflects study questions. CWMs help explain the effects of environmental gradients on community trait composition, whereas the effects of traits on species' niches are better estimated using hierarchical JSDMs.

## Graphical abstract

Trait–niche relationships can provide important insights into community ecology, but several common metrics for these relationships differ subtly. We compare the inferences drawn from community‐weighted mean trait values and from trait–niche parameters in joint species distribution models for forest beetle communities in Finland.

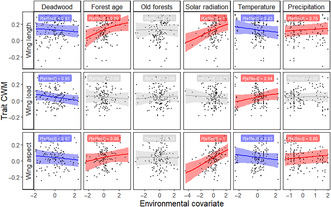

## 1 INTRODUCTION

Functional traits are phenotypic characteristics that shape the responses of species to their environment (response traits) or that determine the effects of species on ecosystem functions (effect traits; Díaz et al., 2013; Lavorel, 2013; Violle et al., 2007). Loss of functional diversity and structure can have larger effects on ecosystems and their functions than loss of species alone (Cadotte et al., 2011; Flynn et al., 2009; Mouillot et al., 2013). Information on trait composition of communities can thus provide ecological insights into processes shaping community assembly more effectively than information on taxonomic diversity (Abrego et al., 2017; Perović et al., 2015). Accounting for species traits can also improve predictions of species occurrences (Öckinger et al., 2010), enable generalization across spatial scales (Carmona et al., 2016), provide insight into species interaction networks (Wende et al., 2017), and show how community assembly changes across environmental gradients (Pavoine & Bonsall, 2011).

Environmental filters change species assemblages by benefitting or excluding species with particular traits (Simons et al., 2016). In the Anthropocene, these filters are often shaped by human activities, and effects can be observed both in community trait means and in the variation in trait values in communities. For example, forestry intensification decreases the relative abundance of especially large‐bodied beetles that depend on large tree trunks (Seibold et al., 2015), and reduces trait variability in forest beetles (Gossner et al., 2013). Humans further modify landscapes by fragmenting habitat (Haddad et al., 2015), changing habitat patch size as well as local conditions, such that some species struggle to persist (Foley et al., 2005; Luther et al., 2022). Species with stronger dispersal ability are often less affected by fragmentation (Bouget et al., 2015; Van Dyck & Matthysen, 1999). However, in boreal forests, forestry intensification simplifies forest structure (Gauthier et al., 2015) and modifies stand micro‐climates due to increased sun and wind penetration (Greiser et al., 2018). These changes in local‐ and landscape‐scale forest conditions represent changed environmental filters that potentially affect the community trait compositions of forest communities, impacting ecosystem functions.

Effects of environmental conditions on the trait composition of communities have been shown in many systems, and the number of studies addressing trait relationships for insects is growing. Traits like dietary breadth, dispersal ability, voltinism, and body size have been related to the response of insects to habitat structure or disturbance (Didham et al., 1998; Driscoll & Weir, 2005; Schweiger et al., 2005; Steffan‐Dewenter & Tscharntke, 2000). However, as recently summarized by Brousseau et al. (2018), studied insect traits are often poorly related to ecological functions and the absence of clearly postulated hypotheses on how traits relate to environmental conditions hinders the generalizations for whole species groups. Therefore, Brousseau et al. proposed a step‐by‐step protocol to evaluate response traits by (1) identifying constraining environmental filters, (2) relating an ecological function to these filters, and (3) justifying the usage of specific traits in relation to this function. Testing relationships between individual traits and species responses to environmental conditions (i.e., their niches), each representing a trait–niche relationship, can thus help to identify appropriate set of traits for future generalizations across taxa and to understanding mechanisms behind these relationships. This is especially important as many potential trait–niche relationships are untested, and contrasting arguments on how trait composition relates to environmental gradients can often be made (Brousseau et al., 2018).

Which measure(s) should be used to study trait–niche relationships is debated (Brousseau et al., 2018; Miller et al., 2019; Muscarella & Uriarte, 2016; Peres‐Neto et al., 2017). The most common measure, the community‐weighted mean (CWM) trait value (Shipley et al., 2006), is weighted by species abundance/prevalence. If common species drive ecosystem functions, CWMs may therefore be the metric of choice for studies relating environmental change to community functional change. Yet rare species, although poorly known and sampled (Burner, Birkemoe, et al., 2022), can also be important to ecosystem function (Burner, Drag, et al., 2022; Dee et al., 2019; Mouillot et al., 2013; Simpson et al., 2022), necessitating a measure that better explores the links between species' traits and their niches.

An alternative to CWMs is to model species' responses to the environment (i.e., their niches) as a function of their traits using hierarchical multispecies joint species distribution models (JSDMs), which estimate the influences of species traits on niches simultaneously while estimating those niches (Ovaskainen et al., 2017). These models include regression coefficient estimates, designated Γ, for each species trait–environmental covariate pair that indicate how a change in trait value would influence a species' response to that covariate. For example, a positive value for the Γ parameter representing the relationship between wing length and forest age would indicate that species with longer wings response positively to older forests relative to shorter winged taxa.

When estimating Γ (and unlike for CWMs), no weighting by abundances/prevalence is performed, although in practice the rarest species are often excluded, and common species can exert more influence because uncertainty in their estimated niches is typically low relative to less common species. The focus of the Γ measure, however, is on the functional relationship between species' traits and their niches. Whereas CWM trait values demonstrate the role of the environment in shaping trait values in realized assemblages, trait‐informed Γ parameters in JSDMs test for generalizable patterns in how traits affect the distributions of individual species, whether rare or common. This JSDM trait metric, important for studies of community functional structure in situations where less common species play a role (Dee et al., 2019), is also helpful in ecological studies where the primary interest is in determining the link between species traits and their niches. However, we are not aware of studies comparing conclusions drawn from these two measures.

Deadwood‐dependent (i.e., saproxylic) beetles represent a species‐rich community important for ecosystem functions, such as wood decomposition and carbon fluxes (Seibold et al., 2021; Stokland et al., 2012). Several of their morphological traits have been suggested to be linked with ecological functions (Hagge et al., 2021), and a wide variety of life histories and ecological traits exist between species. For example, potential dispersal distances may vary from one to tens of kilometers (Komonen & Müller, 2018; Ranius et al., 2019), which may have implications for the species ability to persist in fragmented landscape. However, the links between specific traits, their functions, and important environmental filters remain relatively unexplored (but refer to Burner, Stephan, et al., 2021; Drag et al., 2023; Neff et al., 2022). It was recently demonstrated that rare species may be more important than abundant species for the total functional structure of saproxylic beetles (Burner, Drag, et al., 2022), emphasizing the importance of rare species when studying their functions. This makes saproxylic beetles a good study system for comparing metrics of trait–niche relationships.

We hypothesize that the dispersal function in beetles is constrained by environmental conditions and dependent on species traits (Bouget et al., 2015). Dispersal is a key population process determining species persistence in both natural and fragmented landscapes. Morphological traits have been linked to dispersal ability in several insects, including butterflies (Berwaerts et al., 2002; Sekar, 2012) and stoneflies (McCulloch et al., 2017). However, our general understanding of insect dispersal is sparse, albeit somewhat studied for species of conservation interest or pest species (Feldhaar & Schauer, 2018). For saproxylic beetles, population genetic analyses and direct studies (radiotelemetry and mark–recapture) show a wide range of dispersal distances (Drag et al., 2011; Drag & Cizek, 2018; Komonen & Müller, 2018; Ranius et al., 2019) but are based only on a few model species. An alternative approach is to use several flight‐related morphological traits as proxies for dispersal ability and examine the relationship between these traits and species assemblages across environmental gradients. This indirect approach allows making inferences on trait–niche relationships for an entire insect group. Wing morphology is tied to dispersal ability in many species (Arribas et al., 2012; Gibb et al., 2006; Kobayashi & Sota, 2019), including saproxylic beetles (Jonsson, 2003). Good dispersers are often characterized by having long wings relative to their body size (Southwood & Henderson, 2009), low wing load (mass divided by wing area; Wainwright & Reilly, 1994), and high wing aspect ratio (wing length divided by wing width), indicating high flapping frequency (Hassall, 2015; Norberg, 2012).

The overall aims of this study were to test trait–niche relationships for consistency with hypothesized dispersal effects in boreal saproxylic beetles and to investigate how inferences about the trait composition and niches of species in communities are affected by the metric used to estimate these relationships. To do this, we developed a set of preliminary ecological hypotheses (Table 1), as recommended by the framework of Brousseau et al. (2018). Our environmental predictors included local‐ and landscape‐scale forest covariates affected by industrial forestry (Uhler et al., 2021), as well as climatic covariates (De Kort et al., 2020; Müller et al., 2015). Putative morphological dispersal traits included relative wing length, wing load, and wing aspect ratio. We used JSDMs to estimate the relationships between these traits and a suite of environmental gradients. We then used these models to estimate predicted CWM trait values across these same gradients. Specifically, we asked:

1. Are estimates of the trait–niche relationships in beetles, based on JSDMs, consistent with our dispersal hypotheses (Table 1)?
2. How do these trait effects manifest themselves in local beetle assemblage CWM trait values, and are these CWM values consistent with our hypotheses?
3. What are the implications of using JSDM‐based community‐wide trait–niche relationships versus CWMs in studies of functional structure?

**TABLE 1:** Summary of hypothesized links between dispersal traits and environmental covariates, and their statistical support in our study.

Hypothesis	Examples of support in the literature	*Increase in … leads to*	*Increasing proportion of species with…*	*Trait*	Expected trait–niche relationship	Community response: Γ			Community response: CWM		
						Negative	No	Positive	Negative	No	Positive
Increasing deadwood (local resource amount) and forest age (time available for local population colonization) **increases** the relative frequency of species with **poor dispersal abilities**	Old, deadwood‐rich forest provide long‐lasting and stable habitats that support species with limited dispersal abilities (while ephemeral habitats support species with high dispersal abilities; Feldhaar & Schauer, 2018)	Deadwood (local)	Low	Wing length	Negative		65		81		
			High	Wing load	Positive	80			95		
			Low	Wing aspect	Negative	83			87		
		Forest age	Low	Wing length	Negative			93			99
			High	Wing load	Positive		58			68	
			Low	Wing aspect	Negative		56				88
Increasing old forests (habitat amount in the surrounding landscape) **increases** the relative frequency of species with **poor dispersal abilities**	Increasing colonization rate with increasing connectivity (Hanski, 1999) and mass effect (immigration of individuals from source populations; Leibold et al., 2004) are particularly important for beetles with poor dispersal abilities (Hendrickx et al., 2009)	Old forests in the landscape	Low	Wing length	Negative		51			52	
			High	Wing load	Positive		59			69	
			Low	Wing aspect	Negative		74			53	
Increasing solar radiation and temperature facilitates dispersal, hence **increasing** the relative frequency of species with **poor dispersal abilities**	Specialist saproxylic beetles responded positively to increasing temperature (Gough et al., 2015; Müller et al., 2015). Increasing temperatures increase movement rates and dispersal activity (Jackson et al., 2009; Moser & Dell, 1979).	Solar radiation	Low	Wing length	Negative		65				100
			High	Wing load	Positive	93				71	
			Low	Wing aspect	Negative			85			100
		Temperature	Low	Wing length	Negative		71		83		
			High	Wing load	Positive			88			94
			Low	Wing aspect	Negative		69		83		
Increasing precipitation dampens dispersal, hence **decreasing** the relative frequency of species with **poor dispersal abilities**	Specialist saproxylic beetles in hollow oaks responded negatively to increasing precipitation, although some generalists did not (Gough et al., 2015). Rain dampens dispersal as emergence from host tree and dispersal occur in drier weather conditions (Moser & Dell, 1979)	Precipitation	Low	Wing length	Positive		64				78
			High	Wing load	Negative			86		66	
			Low	Wing aspect	Positive		74				86

*Note*: The columns written using italics translate the general hypotheses into expected relationships within the community for each trait–niche relationship tested; they can be read as, for example, “An increase in deadwood leads to increasing proportion of species with low wing length.” The expected trait–niche relationship reflects the hypothesized direction of community response, as estimated using the two measures (marginal community‐weighted mean (CWM), and Γ). Shown are the estimated Bayesian posterior supports for each relationship, which are further grouped as positive (>75% support), no supported response, or negative (>75% support). Shaded cells show expected relationships.

Bold text in the hypothesis column shows the direction of the hypothesized effects of each covariate on beetles with putative poorer dispersal abilities.

## 2 MATERIALS AND METHODS

### 2.1 Study area and beetle sampling

**FIGURE 1:**
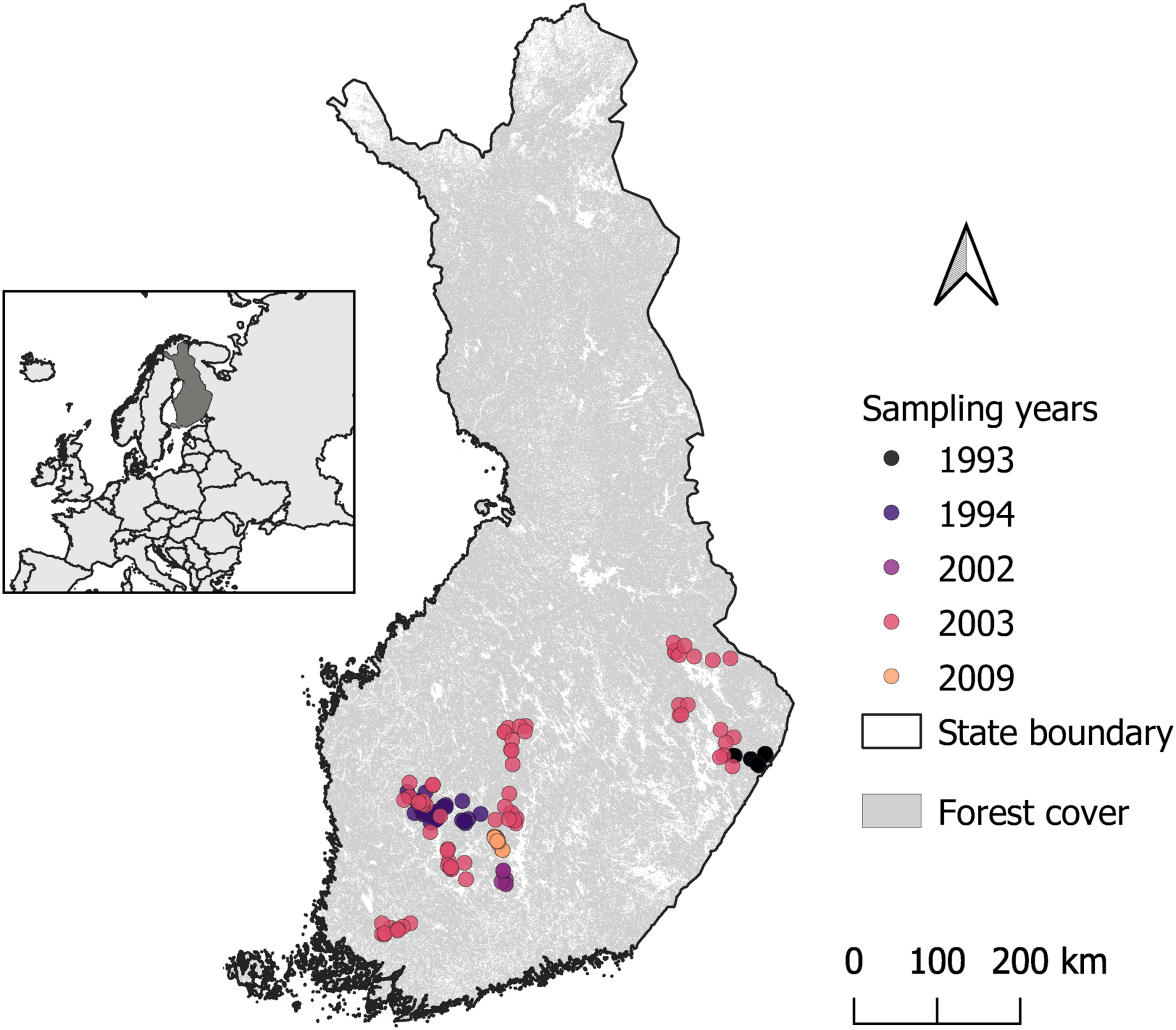
Location and year of beetle sampling at the 142 study sites in Finland.

Beetle sampling was conducted between 1993 and 2009 across the southern and middle boreal vegetation zones in Finland (Figure 1; Figure S1). All sites (*n* = 142) were located within forest stands that were managed, seminatural or natural closed‐canopy forests of different successional stages. Most of the forests were dominated or admixed by Norway spruce (*Picea abies*) or Scots pine (*Pinus sylvestris*). Within each site, window traps were suspended on a string between tree trunks, most often of Norway spruce (Burner, Birkemoe, et al., 2021), about 1 m above the ground. Each trap was made of two perpendicular, intercepting 40 by 60 cm transparent plastic panes. At each site, five traps were used, except in 2009 (10 traps) and in 2002 (six traps). Traps were deployed between May and September with collection bottles containing water, salt (NaCl) as a preservative, and several drops of odorless, allergy‐free detergent. Beetles were collected from traps three to five times during this period, depending on the year. We pooled the individuals from all traps and empty periods at a site within a year for modeling. Further sampling details are available in previous publications (Jokela et al., 2018; Martikainen et al., 1996, 2000; Nordén et al., 2013; Siitonen, 2001; Siitonen et al., 2009). Species were identified morphologically by an expert taxonomist and designated as saproxylic based on the German reference list of saproxylic beetles (Köhler, 2000; Schmidl & Bußler, 2004). Species absent from the German list were designated as saproxylic using expert knowledge in Finland. Of 369 saproxylic species captured, we lacked trait information (described below) from 44 of them, which we excluded. Additionally, species with low prevalence (occupying fewer than five sites) were removed prior to modeling because estimating niches with so few detections is usually not possible (Ovaskainen & Abrego, 2020), leaving 212 species for modeling (refer to Table S1 for species list, based on taxonomy of GBIF Secretariat, 2022).

### 2.2 Forest and climatic conditions

For each site, environmental conditions (covariates) at the stand and landscape scale were measured (Figure S1). At the stand scale, stand age (hereafter Forest age, in years) was measured as the mean age of the five oldest trees in the stand. We excluded forest stands with ages of <16 years because of evidence for beetle community dynamics in recent clear‐cuts and other disturbed sites that differ from those in forests (Burner, Birkemoe, et al., 2021; Nilssen, 1984). Within each stand, the total pooled volume of local standing and fallen dead trees (hereafter Deadwood, m^3^/ha) with a minimum diameter of 10 cm was estimated using transects.

At the landscape scale, we also quantified the amount of old forest present in the area around each sampling location. Old forest is deadwood rich, and deadwood is a key resource for saproxylic species (Gibb et al., 2013), but we lacked direct measurements of landscape‐scale deadwood. However, deadwood has been found to increase with forest age and the volume of living trees in a stand (Jacobsen et al., 2015), so we used living volume of old forests as a proxy for landscape‐scale deadwood. To quantify the amount of such habitat in the surrounding landscape, we calculated the volume of living wood in those forests older than 100 years within a 1 km radius around each site (hereafter Old forests, m^3^). We chose 100 years because younger managed forests typically have much less deadwood than older forests in Fennoscandia (2–10 m^3^/ha compared to 60/90 m^3^/ha, respectively; Siitonen, 2001). We chose 1 km because it is assumed that many saproxylic insects can readily colonize substrate within 1 km (Jonsell et al., 1999) and because this makes our results comparable to other studies (e.g., Jacobsen et al., 2020). We calculated these values following the procedure in Mair et al. (2018) using the site centroid coordinates and the multisource National Forest Inventory of forest volume in Finland, downloaded from the Natural Resource Institute Finland (LUKE; http://kartta.luke.fi/opendata/valinta‐en.html, raster resolution of 20 m). We used the data from 2009 (Tomppo et al., 2019) as they are closest in time to the collection year of most beetle data (Figure 1). Additionally, we tested a 5 km radius for calculating these values but found these data to be highly correlated with the 1 km data.

We extracted historical data on temperature (°C) and precipitation (mm) from the ERA5 climate reanalysis (Muñoz‐Sabater et al., 2021) at ~30 km^2^ resolution, rounded to the nearest 0.25′ as calculations are made on a 0.25′*0.25′ grid and total solar radiation (calculated using ArcGIS 10.8) for 1 April to 30 September for each year and location. For temperature, we used estimates of 2 m aboveground temperature and calculated the mean among hourly estimates (24 h/day) for the entire period. For precipitation, we summed all hourly estimates (24 h/day) of precipitation at ground level. For solar radiation, we calculated the sum of all daily values (Wh/m^2^) at each site using ArcGIS. Contrary to the other climatic conditions, solar radiation is not only affected by geographic location but also by topographic aspect and hence describes local thermal conditions at a finer scale.

We assumed that species respond more strongly to equivalent changes in deadwood volume when deadwood is scarce (e.g., 10–15 m^3^/ha deadwood) rather than abundant (e.g., 100–105 m^3^/ha; Martikainen et al., 2000; Müller et al., 2015). Therefore, deadwood (plus a constant of one) and Old forest covariates were log‐transformed. This also avoided high leverage of the largest values in our results. All environmental covariates were centered and standardized (*z*‐scores).

### 2.3 Beetle traits

We used three morphological traits from the trait database in Hagge et al. (2021) that are associated with dispersal abilities: relative wing length, wing aspect ratio (i.e., shape), and relative wing load. Wing length is often standardized by body length because relative, rather than raw, wing length is a better measure of flying ability (Hagge et al., 2021; Kilmer & Rodríguez, 2017). Similarly, wing load is often divided by body length to remove the effects of body length, with which it is highly correlated (Hagge et al., 2021). We thus divided wing length and wing load by body length and then log‐transformed these traits as well as wing aspect. Species with similar mass and wing area (i.e., similar wing load) can have broad, short wings or long, narrow wings (i.e., different aspect ratios), highlighting the importance of wing shape as well as size. Predictions of flight performance ideally would therefore consider all of these traits (Le Roy et al., 2019); hence, we included all of them. Each trait was then centered and standardized (*z*‐scores). We did not use body size (mass and body length) itself as it is a multidimensional functional trait describing many aspects of species biology. For example, large species may also have long generation time and low population density, as well as large home ranges (McKinney, 1997).

### 2.4 Statistical modeling

To estimate trait–niche relationships and CWM trait values, we used joint species distribution models from the hierarchical modeling of species communities (HMSC) R‐package (Tikhonov et al., 2020). This is a multivariate, hierarchical generalized linear mixed model that is fitted to a matrix of species data recorded at each site. The model is fitted using Bayesian inference, thus allowing estimates of the degree of belief in the relationships found. Because species abundance offered little variability, we modeled species presence‐absence values as response data. As predictors, we included explanatory environmental covariates (described below) and modeled species responses to these fixed effect covariates (β_Species, Covariate_) as a function of species traits (refer to Figure S2 for model structure). Our model thus also included a hierarchical level with parameters (Γ_Trait, Covariate_) that describe the influence of traits on species' responses to the environment (i.e., trait–niche relationship).

Additionally, our models included random effects to account for spatiotemporal aspects of our study design. A random effect of year accounts for interannual variability in species communities not accounted for by the included environmental predictors, and a random effect of climate grid cell accounts for instances where multiple sampling locations occurred in the same grid cell of the climate data spatial resolution. Finally, a random effect of sampling unit (site) was included to incorporate species co‐occurrence structure (estimated using latent variables; Ovaskainen et al., 2016). This random effect was spatially structured and so also accounted for spatial autocorrelation. We modified the default prior of this spatial random effect to consider distance‐based correlations in community composition up to 170 km pairwise distance (rather than the maximum pairwise distance in the dataset of 503 km) because a range of distances above 170 km seldom occurred in the dataset (Figure S3). We further included a phylogenetic correlation matrix in the covariance structure of the model to determine whether residual variation in species niches (not explained by traits) was phylogenetically correlated (parameter *ρ*). A *ρ* close to one indicates that this residual variation in niches is highly phylogenetically correlated, whereas a value close to zero indicates a lack of such correlation. We did this using a species‐level phylogeny with branch lengths based on Chesters (2017) as used by Burner, Stephan, et al. (2021). Species included in our final dataset but missing from this phylogeny were added randomly to the proper genus or, occasionally, family.

Our six environmental covariates (Figure S1) were modeled as fixed effects. To account for the differing sampling effort, we also included the log of the number of traps at each site as an additional fixed effect (Ovaskainen & Abrego, 2020). Because precipitation and temperature were highly correlated (−0.78), and we were interested in independent effects of both, each was included in a separate model that included all other covariates. All covariates in each model had variance inflation factors (VIF) of less than three, indicating low multicollinearity. Except for precipitation‐specific estimates, all parameter estimates are presented from the temperature model (Model 1). The explanatory power of each model was quantified using the average AUC (area under the curve) and Tjur *r* ^2^ (Tjur, 2009) values across all species. We further used variance partitioning to estimate the percent of variance explained by each environmental covariate and what percent of responses to covariates ($r_{\text{Niche}}^{2}$) and overall occurrence patterns ($r_{\text{Occs}}^{2}$) were explained by traits.

Models were fitting using Markov chain Monte Carlo (MCMC) with three chains, each run for 6000 iterations with 2500 discarded as burn‐in. The remaining iterations were thinned by 10 to yield 350 samples per chain (1050 total). Default prior distributions were used, except for the spatial random effect (described above) and model convergence was examined using the potential scale reduction factors (Gelman & Rubin, 1992) and the effective number of iterations.

### 2.5 Comparing two measures of community trait response

We considered and compared two measures of trait–niche relationships. The first was the JSDM parameter Γ, which shows the relationship between species' traits and β responses. The signs and support levels of these parameters were compared with our preliminary hypotheses (Table 1). Our second measure of trait–niche relationships, CWM, is a classical measure that is calculated for local assemblages by averaging trait values for each species, weighted by the prevalence or abundance of each species. To estimate CWMs, we used our fitted JSDMs to generate 1050 predicted beetle communities (one per MCMC sample) for each of 20 covariate values across a gradient of each environmental covariate. CWM trait values were then calculated (with associated credible intervals), weighted based on species prevalence across all predicted communities at a given point on each gradient.

As the rarest species were excluded to enable model convergence, we evaluated the effects of this removal on CWM values. To do this, we first tested the relationship between raw CWMs at our sampled sites for all species for which trait information was available (*n* = 325; described above) versus the modeled species (*n* = 212). They were highly correlated (*r* ^2^ > .98; Figure S4), providing evidence that our inferences from model‐estimated CWMs across gradients should hold for the communities as a whole (including the less common species).

To quantify the probability of an increase or decrease (posterior support) of the CWMs across each gradient in covariate values, we calculated the difference between the CWMs predicted for communities at lowest and highest ends of each focal environmental covariate gradient using predicted communities based on each MCMC sample. We present the marginal relationships (main results), in which nonfocal environmental covariates are kept at their global means across gradients in each focal covariate, as well as the total (net) relationships (Supporting Information), in which the nonfocal covariates are set to their most likely values across each focal covariate gradient based on their linear relationship with the focal variable (Ovaskainen & Abrego, 2020). Finally, we compare the estimated relationships revealed by CWMs and Γ to our hypotheses and to each other.

## 3 RESULTS

The community models explained, averaged over all species, 13% of the variation in species occurrences (Table 2). Individual forest and climate covariates explained 6%–12% of the variability in species occurrences, based on variance partitioning. Temperature explained somewhat more variation than precipitation, although the two models were similar overall.

**TABLE 2:** Amount of variance in species occurrences explained by environmental covariates and random effects.

Model	Deadwood	Forest age	Old forests	Solar radiation	Temperature	Precipitation	Effort	Spatial site	Random year	Random climate ID	AUC	Tjur *r* ^2^
1	0.100	0.104	0.066	0.060	0.117	–	0.161	0.155	0.126	0.111	0.810	0.132
2	0.102	0.118	0.071	0.059	–	0.085	0.163	0.150	0.143	0.110	0.812	0.133

*Note*: Models are identical except that they include temperature or precipitation covariates, respectively. For the seven fixed effects and three random variables, the percent of variance explained is shown. The explanatory power (Tjur *r* ^2^, AUC) is averaged across all species.

### 3.1 The importance of dispersal traits in explaining species niches

Wing load, aspect, and length together explained 3%–12% of the variance in relationships between occurrences and environmental covariates ($r_{\text{Niche}}^{2}$; Table 3) and 2.6% of the total variance in species occurrences ($r_{\text{Occs}}^{2}$). Related species responded similarly to the environmental covariates, as indicated by the moderate phylogenetic signal in species niches (*ρ*; Table 3).

**TABLE 3:** Amount of variance in species occurrences and niches explained by species traits.

Model	$r_{\text{Occs}}^{2}$	*ρ*	$r_{\text{Niche}}^{2}$ Deadwood	$r_{\text{Niche}}^{2}$ Forest age	$r_{\text{Niche}}^{2}$ Old forests	$r_{\text{Niche}}^{2}$ Solar radiation	$r_{\text{Niche}}^{2}$ Temperature	$r_{\text{Niche}}^{2}$ Precipitation	$r_{\text{Niche}}^{2}$ Effort
1	0.026	0.54–0.77	0.043	0.079	0.026	0.124	0.043	–	0.016
2	0.027	0.54–0.78	0.044	0.077	0.025	0.147	–	0.028	0.012

*Note*: Models are identical except that they include temperature or precipitation covariates, respectively. Shown are percentage of the variance in occurrences explained by the combination of all three traits ($r_{\text{Occs}}^{2}$), the signal of a phylogenetic effect in species responses to their environment (*ρ*; 95% credible intervals), and percentages of variance in relationships between species occurrence and environmental covariates (their niches) explained by the traits ($r_{\text{Niche}}^{2}$ for each environmental covariate).

### 3.2 Evaluating support for hypotheses

**FIGURE 2:**
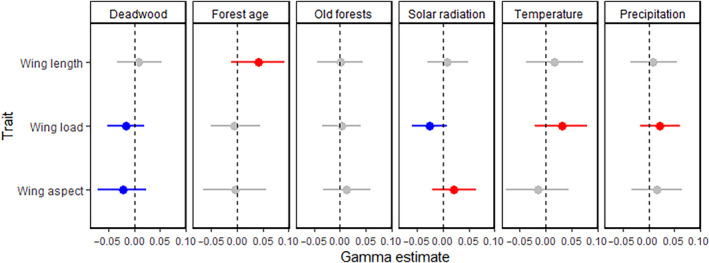
Effect of dispersal traits on the estimated relationship between species occurrence and environmental covariates. These Γ estimates (*x*‐axes) indicate whether species with larger values of a given trait (*y*‐axis) respond positively or negatively to each environmental covariate (plot facets), relative to species with smaller trait values. Whiskers show 95% credible intervals (CIs), and Γ estimates with >75% posterior support are colored red (positive) or blue (negative). Hypothesized relationship between traits and species responses to the environmental covariates presented in Table 1.

Based on JSDM Γ parameters, we found some evidence that dispersal traits predict species' responses to environmental gradients (Figure 2; refer to Figure S5 for species responses to the environment, i.e., *β*). Out of the 18 investigated relationships, only one had >95% support although seven showed signs that traits influenced species niches (>75% support). These seven potential relationships were consistent with only two of our 18 dispersal‐driven preliminary ecological hypotheses (Table 1; >75% support) but were opposite to our prediction for five hypotheses.

**FIGURE 3:**
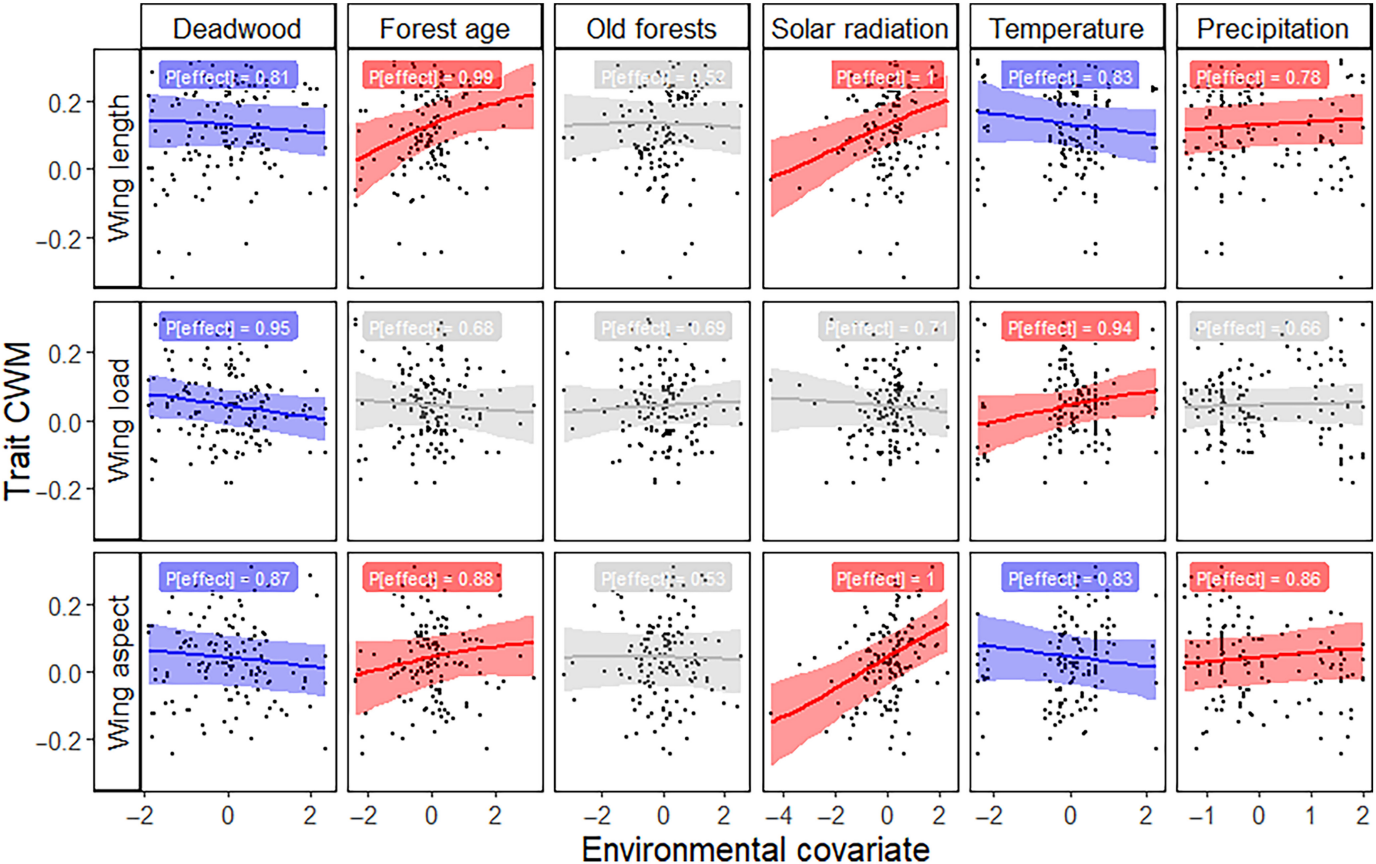
Predicted community‐weighted mean (CWM) trait values across environmental gradients. Lines show the posterior mean of marginal effects (with 95% credible intervals). Nonfocal environmental covariates were held at their mean (0) across the focal variable gradients (*x*‐axis; to show marginal effects). Points show the CWM at each site. CWM estimates with >75% posterior support are colored red (positive) or blue (negative), with values at the top of each facet showing posterior support. For total (net) effects, refer to Figure S6. For the relationship between CWM and Γ probabilities, refer to Figure S7.

Based on the model‐predicted CWM trait values, environmental covariates also affect the dispersal trait composition of saproxylic beetle communities (Figure 3). Out of the 18 trait–niche relationships, 12 showed signs of an increase or decrease in the marginal CWMs along the environmental gradients (>75% support), with four trait–niche relationships being highly likely (>95% support). For net (total), rather than marginal, effects refer to Figure S6. These relationships were consistent with seven of our preliminary hypotheses (Table 1; >75% support) but were opposite our predictions in five cases.

### 3.3 Comparing community response measures

Comparing JSDM Γ parameters and CWMs, CWMs showed a larger number of supported relationships in our dataset (Table 1, Figures 2 and 3). Of seven Γ relationships with moderately high support (>75%), CWMs showed effects in the same direction for five of them and showed no effect for the other two. However, CWMs were more likely to show effects and had stronger support on average, revealing an additional seven supported relationships between communities and environmental covariates not predicted by Γ. Nevertheless, probabilities of positive and negative relationships were correlated between the two measures (*r* ^2^ = .48; Figure S7).

## 4 DISCUSSION

We developed a set of 18 preliminary hypotheses linking beetle dispersal traits with ecological gradients pertaining to habitat fragmentation in a managed forest landscape in Finland. We found that traits explain variation in species' realized niches and that forest and climatic conditions act as environmental filters that change the dispersal trait composition of beetle communities. However, the direction of these effects was seldom as predicted by our hypotheses. Furthermore, we compare the inferences using a standard method (trait community‐weighted means; CWMs) with those revealed by hierarchical joint species distribution models (Γ parameters). CWMs showed often more highly supported (and sometimes opposite; Table 1) effects than did Γ, highlighting differences in the two methods.

Up to 12.4% of the beetle responses to a given environmental covariate could be explained by our three morphological dispersal traits (Table 3). The traits explained more in the responses of the species to forest age and solar radiation than to other covariates, but our hypothesized dispersal traits did not predict species responses to the amount of old forest in the surrounding landscape, which contrasts with previous work that showed an impact of habitat fragmentation on ecological feeding guilds trait distributions (Didham et al., 1996). However, Finnish forests occur primarily in largely forested landscapes, and there is evidence that this makes dispersal limitation less important (Janssen et al., 2016; Seibold & Thorn, 2018), although it does appear to be important in long fragmented areas (Brin et al., 2016) and could indeed be important for certain species (Ranius et al., 2019).

Our findings were consistent with only 11% (Γ) or 39% (CWMs) of our hypotheses (Table 1). This is perhaps not unexpected, because even highly supported trait–niche relationships have been found to lack consistency among regions (Burner, Stephan, et al., 2021). Traits may influence niches differently and relate differently to ecological functions for various subgroups of beetles that have very different life histories and that vary widely in body size and habitat use. Also, taxonomically and ecologically diverse saproxylic beetles are challenging to model using typical SDM covariates that may poorly reflect the micro‐habitat and micro‐climate signals to which they respond (Gossner et al., 2013), although there is evidence that broader‐scale environmental filters also play important roles (Hagge et al., 2019; Neff et al., 2022).

These results highlight the difficulty in linking morphological traits to species niches (Barton et al., 2011; Drag et al., 2023). This is true even when following a recommended hypothesis framework (Brousseau et al., 2018) for an apparently straightforward function (dispersal) and set of traits (wing morphology), using covariates that contribute directly to habitat connectivity. Caution is thus due in attempts to link insect morphology to less straightforward functions, or especially to ecosystem services, except in the case of pollinators where insect and flower morphologies appear to sometimes be closely linked (Mayfield et al., 2001). However, the considerable residual phylogenetic signal in species niches in our models indicates that some unmeasured by phylogenetically correlated trait or suite of traits (Ovaskainen & Abrego, 2020) does have an influence on beetle niches.

CWM trait values are commonly examined because they provide a concise and readily estimated summary of trait values in realized communities (Miller et al., 2019). This property is often a strength when trying to understand how communities respond to environmental gradients and changes (Shipley et al., 2006), but it leads to limitations when CWMs are used to estimate trait–niche relationships. In our study, CWMs detected 50% more apparent trait–niche relationships (>75% support) than did JSDM Γ parameters. This disparity is because common species dominate the CWM responses, whereas Γ effects are more evenly weighted across the community (Ovaskainen & Abrego, 2020), although common species may be more influential because their niches are estimated with more precision. CWM values are thus sensitive to any common species that are outliers (whether in magnitude or direction) in community‐wide trait–niche relationships, as well as to nonindependence in species occurrences (Peres‐Neto et al., 2017; Zelený, 2018), potentially biasing research that attempts to develop generalizations about the link between traits (whether morphological or ecological) and the environment (Fountain‐Jones et al., 2015; Violle et al., 2007). We empirically demonstrate these limitations of CWMs, which have been predicted by theory and produced in silico (Zelený, 2018), by comparing to community‐wide estimates of trait function. Alternative methods commonly used to analyze trait–environment relationships could be further applied (e.g., the fourth‐corner approach; Legendre et al. (1997)). Although this approach has some advantages over CWM (Peres‐Neto et al., 2017), its direct comparison with JSDM has resulted in comparable outcomes (Ovaskainen & Abrego, 2020).

### 4.1 Conclusions

Trait–niche relationships for morphological traits are commonly evidenced by well‐supported relationships, but these often conflict with straightforward hypotheses (as in the present work) or vary in time and space (Burner, Stephan, et al., 2021). Hypotheses‐driven approaches must not be abandoned (Brousseau et al., 2018), but rather must be approached with due caution and humility. We have shown that inferences on trait–environment relationships will differ when alternate measures are used. CWMs remain well suited to understanding the impacts of environmental gradients on realized communities, and in particular on the common species in those communities, if appropriate consideration is given to the effects of species nonindependence (Zelený, 2018). Hierarchical JSDMs that estimate trait–niche relationships for the community as a whole can be best used for understanding the functions of traits in determining the ecology of species and thus also for predicting the niches of rare and poorly known species for which trait information exists. Both metrics could often be present side by side to show distinct but related effects of the connections between traits, niches, and communities, a multi‐inference analog to the ensemble modeling used in many species distribution and climate studies (Hao et al., 2019). Our results highlight that choice of appropriate community trait metric(s) is thus critical and depends on the goals of a study.

## AUTHOR CONTRIBUTIONS

**Ryan C. Burner:** Conceptualization (supporting); formal analysis (equal); methodology (equal); visualization (equal); writing – original draft (equal). **Jörg G. Stephan:** Conceptualization (lead); data curation (equal); formal analysis (equal); methodology (equal); visualization (equal); writing – original draft (equal). **Lukas Drag:** Data curation (supporting); methodology (supporting); writing – review and editing (equal). **Mária Potterf:** Visualization (equal); writing – review and editing (equal). **Tone Birkemoe:** Conceptualization (supporting); funding acquisition (supporting); supervision (supporting); writing – review and editing (equal). **Juha Siitonen:** Data curation (lead); investigation (lead); writing – review and editing (equal). **Jörg Müller:** Conceptualization (supporting); writing – review and editing (equal). **Otso Ovaskainen:** Formal analysis (supporting); methodology (supporting); software (lead); writing – review and editing (equal). **Anne Sverdrup‐Thygeson:** Conceptualization (supporting); funding acquisition (supporting); supervision (supporting); writing – review and editing (equal). **Tord Snäll:** Conceptualization (supporting); funding acquisition (lead); methodology (supporting); project administration (lead); supervision (lead); writing – review and editing (equal).

## CONFLICT OF INTEREST STATEMENT

The authors have no conflict of interest to declare.

## Supporting information


Data S1

## Data Availability

Data used in this study are available from Burner et al. (2023) at https://doi.org/10.5281/zenodo.8322080.
