# Supplementary material for: Alternative measures of trait–niche relationships: A test on dispersal traits in saproxylic beetles
Source: Ecol Evol. 2023 Oct 19;13(10):e10588. doi: 10.1002/ece3.10588 (PMC10585442; doi:10.1002/ece3.10588)
Supplement: Supplementary file 1 — Data S1 [file ECE3-13-e10588-s001.docx]

Supporting information for:

**Alternative measures of trait-niche relationships: a test on dispersal traits in saproxylic beetles**

Ryan C. Burner^1,2,†,^*, Jörg G. Stephan^3,†^, Lukas Drag^4,5^, Mária Potterf^6^, Tone Birkemoe^2^, Juha Siitonen^7^, Jörg Müller^4,8^, Otso Ovaskainen^9,10,11^, Anne Sverdrup-Thygeson^2^, Tord Snäll^3^,

^1^U.S. Geological Survey, Upper Midwest Environmental Sciences Center, La Crosse, Wisconsin, USA

^2^Faculty of Environmental Sciences and Natural Resource Management, Norwegian University of Life Sciences, Ås, Norway

^3^SLU Swedish Species Information Centre, Swedish University of Agricultural Sciences, Uppsala, Sweden

^4^Field Station Fabrikschleichach, Department of Animal Ecology and Tropical Biology, Biocenter, University of Würzburg, Rauhenebrach, Germany

^5^Institute of Entomology, Biology Centre of the Czech Academy of Sciences, Ceske Budejovice, Czech Republic

^6^Department of Life Science Systems, Technical University of Munich, Freising, Bavaria, Germany

^7^Natural Resources Institute Finland (Luke), Helsinki, Finland

^8^Bavarian Forest National Park, Grafenau, Germany

^9^Department of Biological and Environmental Science, University of Jyväskylä, Jyväskylä, Finland

^10^Organismal and Evolutionary Biology Research Programme, Faculty of Biological and Environmental Sciences, University of Helsinki, Helsinki, Finland

^11^Department of Biology, Centre for Biodiversity Dynamics, Norwegian University of Science and Technology, Trondheim, Norway

^†^ Authors contributed equally

* corresponding author: [rburner@usgs.gov](mailto:rburner@usgs.gov)

**ACKNOWLEDGEMENTS**

The study was funded by Formas (2018-02435) and the part of the BiodivERsA project BioESSHealth – Scenarios for biodiversity and ecosystem services acknowledging health" coordinated by TS. Clayton Traylor provided helpful comments on the manuscript. The computations were enabled by resources provided by the Swedish National Infrastructure for Computing (SNIC) at UPPMAX partially funded by the Swedish Research Council through grant agreement no. 2018-05973. Any use of trade, firm, or product names is for descriptive purposes only and does not imply endorsement by the U.S. Government.

**DATA AVAILABILITY STATEMENT**

Data used in this study are available at <https://doi.org/10.5281/zenodo.8322080>

**SUPPORTING INFORMATION**

**Supporting figures**

**
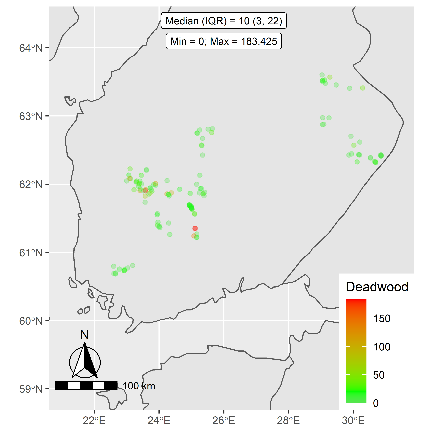

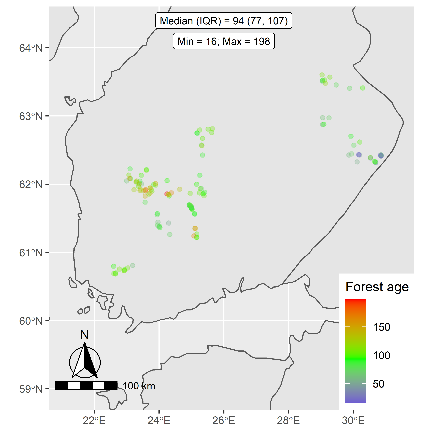

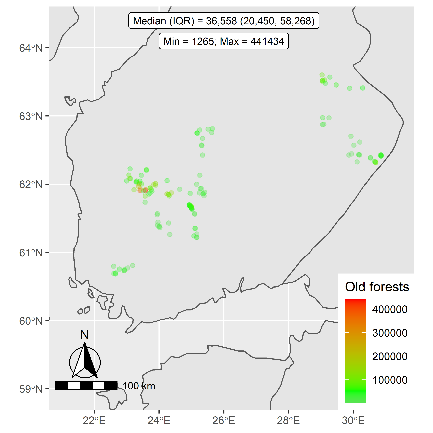
**

**
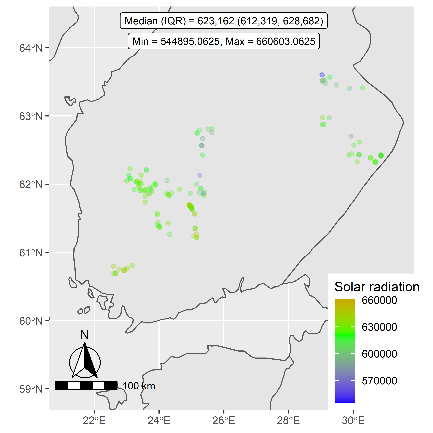

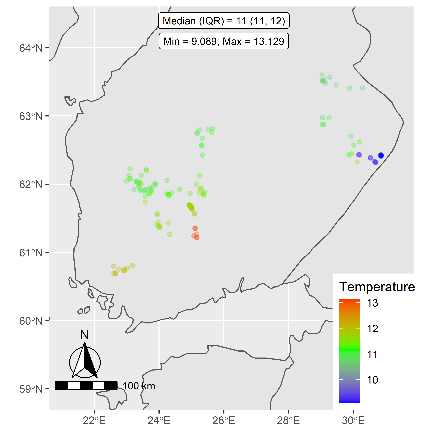

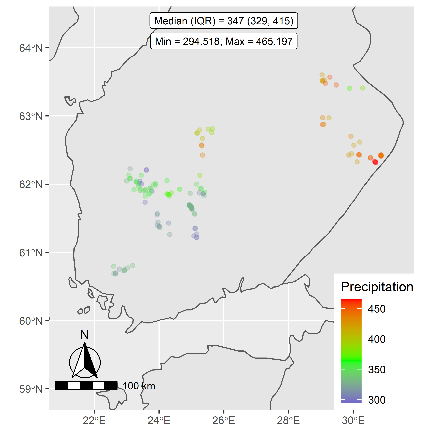
**

**
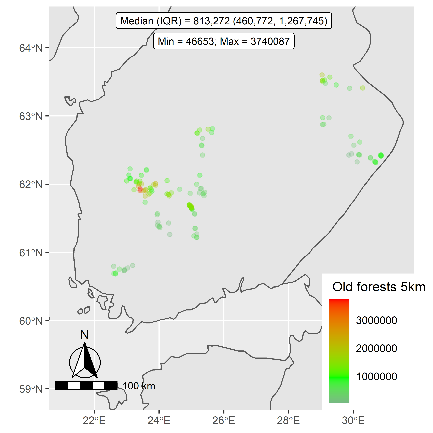

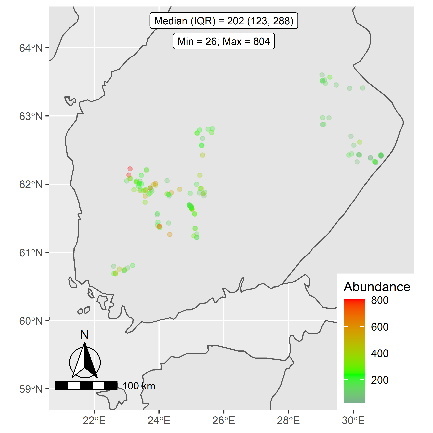

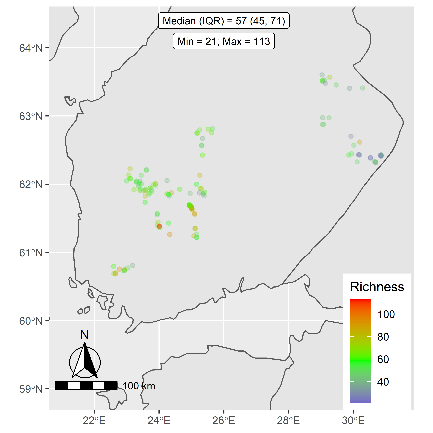
**

**Figure S1.** Spatial distributions of variation in environmental covariates, beetle species richness, and beetle abundance. Deadwood: volume (m^3^/ha) of standing and fallen dead trees with a minimum diameter of 10 cm; Forest age: mean age of the five oldest trees in stand; Old forests: volume (m^3^) of living wood in forest older than 100 years within a radius of 1 km; Solar radiation: sum of daily values (Wh/m^2^) for 1 April to 30 September for each year and location; Temperature: mean (ºC) among hourly estimates (24 hours/day) 2 m above the ground for 1 April to 30 September for each year and location; Precipitation: summed hourly estimates (24 hours/day) of precipitation at ground level for 1 April to 30 September for each year and location; Old forests 5km: volume (m^3^) of living wood in forest older than 100 years within a radius of 5 km; Abundance/Richness of saproxylic beetles trapped at each site.


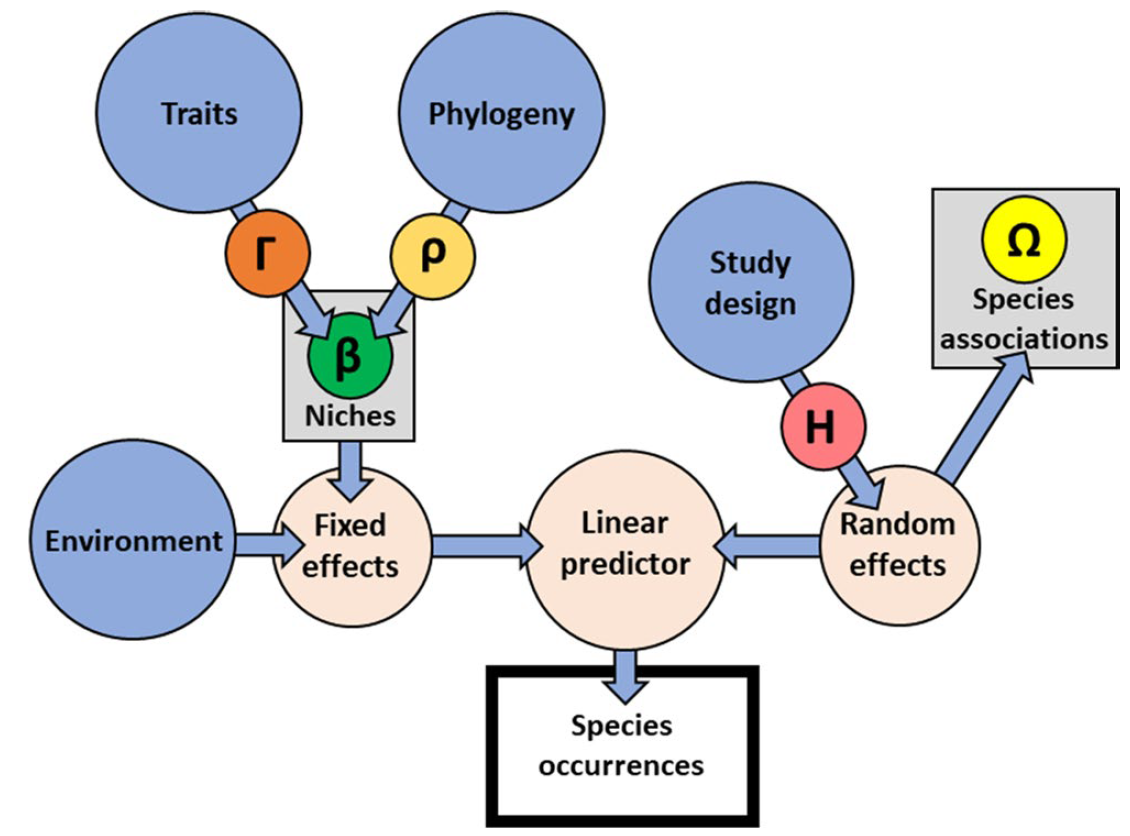


**Figure S2.** Model structure of joint species distribution model performed with the Bayesian hierarchical modeling of species communities (HMSC) (Ovaskainen & Abrego 2020). Blue circles signify data and Greek letters model parameters. Species niches (β) are modeled as a function of species’ shared traits and phylogeny with associated parameters (Γ, ρ) estimating these relationships. Residual associations among species (Ω) that are not explained by species niches are estimated using a latent variable structure. Random effects in our model, here indicated by a study design (Η), were Year and Climate grid cell, as well as each Site with their coordinates (spatial random effect). Figure modified from Burner *et al.* (2021).


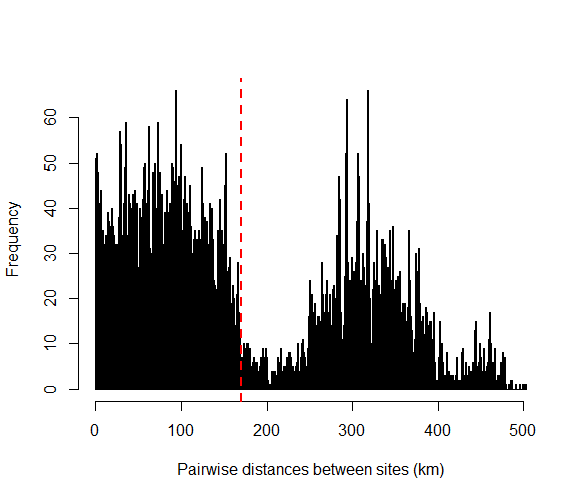


**Figure S3**. Distribution of inter-site distances in the data. The spatial effect prior in the model was modified to consider distances up to 170 km (dashed red line) rather than across the entire range of inter-site distances due to gaps in the histogram at greater distances.


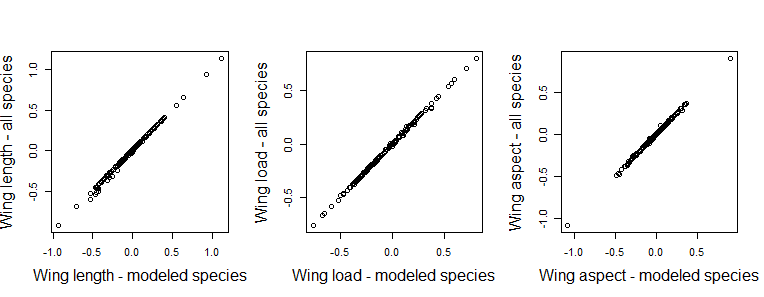


**Figure S4**. Comparisons of beetle CWM trait values from sites sampled. Compares CWM calculated from all species with >=5 detections in the total dataset (x-axis; those used for modeling; n = 212) and all species for which trait information was available (y-axis; n = 325 of 369 saproxylic species detected).

**~~
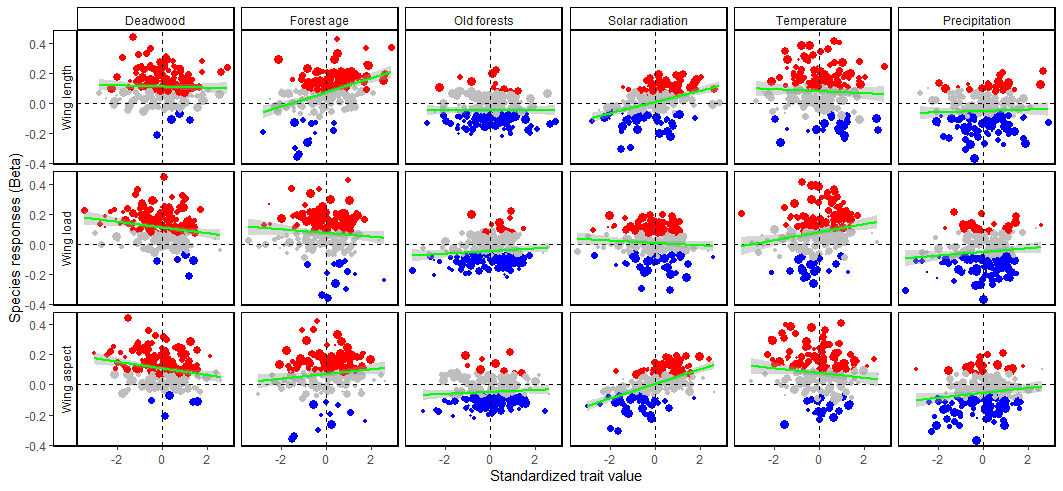
~~**

**Figure S5**. Estimates of relationships between species occurrences and the environmental conditions (β parameters) shown in relation to species trait values. Positive β estimates are red and negative are blue (>75% posterior support. Circle area is proportional to the species’ log-transformed prevalence. Species above/below the horizontal broken line show a positive/negative response to the environmental condition. Species left/right of the vertical broken line have lower/higher than average trait values. Green regression lines show best fit relationships between species traits and β estimates; for Γ parameter estimates quantifying these relationships, and their posterior support, refer to Fig. 2.

**
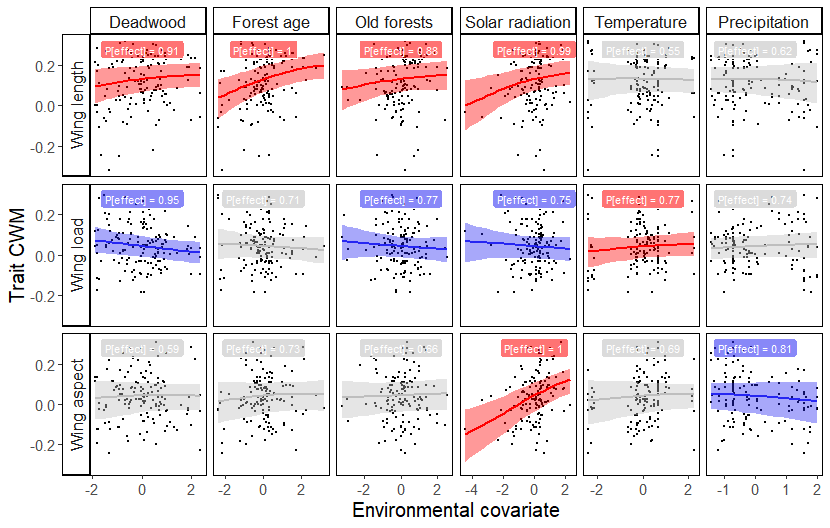
**

**Figure S6.** Predicted community weighted mean (CWM) trait values across environmental gradients. Lines show the posterior mean of total (net) effects (with 95% credible intervals). Non-focal environmental covariates were varied across the focal variable gradient as predicted by a linear model estimating their relationship with the focal variable (x-axis; to show total net effects). Points show the CWM at each site. CWM estimates with >75% posterior support are colored red (positive) or blue (negative), with values at the top of each facet showing posterior support. For marginal effects, refer to Fig. 3.


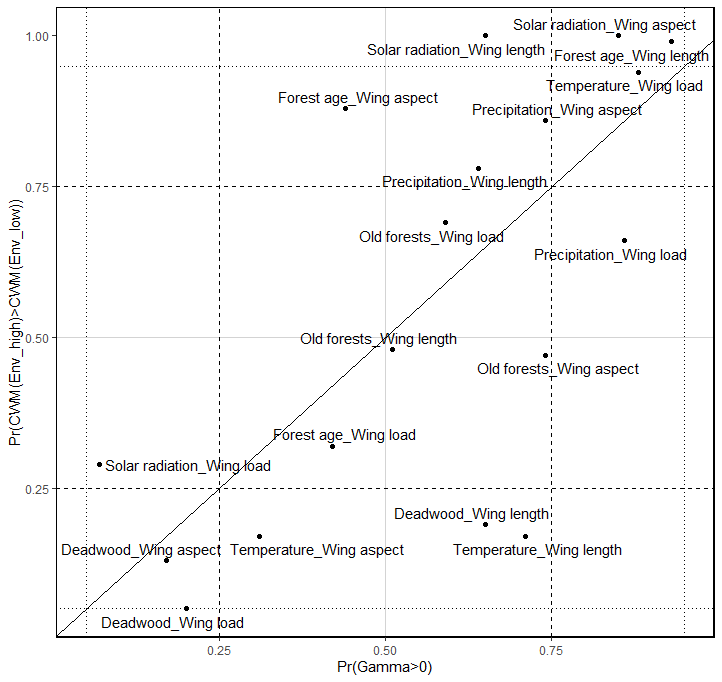


**Figure S7.** Comparison of posterior support values for two metrics of effects of traits on species responses to covariates. X-axis shows posterior probability of a positive Γ (Gamma) parameter estimate from our HMSC models. Y-axis shows probability that a CWM trait value at the high end of a predicted environmental gradient that is higher than at the low end, based on model predictions. Points are labeled with the covariate-trait relationship that they represent. Diagonal line shows where values equal on both axes would fall. Dashed lines show relationships with >75% positive or negative (values of <25%) support, and dotted lines show the same with 95% support. Probabilities were correlated between the two measures (r^2^ = 0.46).

**Supporting tables**

**Table S1:** Saproxylic beetle species included in joint species distribution models in Finland. Species names follow the GBIF backbone taxonomy (GBIF Secretariat 2021).

| Family | Species | Sites (n) |
| --- | --- | --- |
| Anobiidae | *Dorcatoma dresdensis* | 19 |
| Anobiidae | *Dorcatoma punctulata* | 14 |
| Anobiidae | *Dorcatoma robusta* | 7 |
| Anobiidae | *Ernobius abietis* | 16 |
| Anobiidae | *Ernobius explanatus* | 5 |
| Anobiidae | *Hadrobregmus pertinax* | 19 |
| Cantharidae | *Cantharis brevicollis* | 130 |
| Cantharidae | *Malthinus biguttatus* | 84 |
| Cantharidae | *Malthinus flaveolus* | 36 |
| Cantharidae | *Malthodes fuscus* | 87 |
| Cantharidae | *Malthodes guttifer* | 103 |
| Cantharidae | *Malthodes mysticus* | 34 |
| Cantharidae | *Malthodes pumilus* | 26 |
| Cantharidae | *Malthodes spathifer* | 26 |
| Cerambycidae | *Alosterna tabacicolor* | 35 |
| Cerambycidae | *Judolia sexmaculata* | 11 |
| Cerambycidae | *Leptura quadrifasciata* | 7 |
| Cerambycidae | *Molorchus minor* | 14 |
| Cerambycidae | *Oxymirus cursor* | 18 |
| Cerambycidae | *Pogonocherus fasciculatus* | 21 |
| Cerambycidae | *Rhagium inquisitor* | 10 |
| Cerambycidae | *Rhagium mordax* | 19 |
| Cerambycidae | *Tetropium castaneum* | 22 |
| Cerambycidae | *Tetropium fuscum* | 5 |
| Cerambycidae | *Vesperus luridus* | 16 |
| Cerylonidae | *Cerylon fagi* | 44 |
| Cerylonidae | *Cerylon ferrugineum* | 69 |
| Cerylonidae | *Cerylon histeroides* | 65 |
| Ciidae | *Cis boleti* | 40 |
| Ciidae | *Cis glabratus* | 29 |
| Ciidae | *Cis jacquemartii* | 21 |
| Ciidae | *Cis lineatocribratus* | 8 |
| Ciidae | *Cis micans* | 34 |
| Ciidae | *Cis punctulatus* | 37 |
| Ciidae | *Dolichocis laricinus* | 7 |
| Ciidae | *Ennearthron cornutum* | 6 |
| Ciidae | *Orthocis alni* | 23 |
| Cleridae | *Thanasimus formicarius* | 10 |
| Cryptophagidae | *Atomaria alpina* | 7 |
| Cryptophagidae | *Atomaria atrata* | 54 |
| Cryptophagidae | *Atomaria bella* | 38 |
| Cryptophagidae | *Atomaria longicornis* | 17 |
| Cryptophagidae | *Atomaria ornata* | 67 |
| Cryptophagidae | *Atomaria subangulata* | 15 |
| Cryptophagidae | *Atomaria turgida* | 61 |
| Cryptophagidae | *Atomaria umbrina* | 9 |
| Cryptophagidae | *Cryptophagus dorsalis* | 24 |
| Cryptophagidae | *Micrambe abietis* | 135 |
| Cryptophagidae | *Micrambe longitarsis* | 88 |
| Cryptophagidae | *Pteryngium crenatum* | 31 |
| Curculionidae | *Crypturgus cinereus* | 8 |
| Curculionidae | *Crypturgus hispidulus* | 41 |
| Curculionidae | *Crypturgus subcribrosus* | 25 |
| Curculionidae | *Dryocoetes alni* | 26 |
| Curculionidae | *Dryocoetes autographus* | 139 |
| Curculionidae | *Dryocoetes hectographus* | 64 |
| Curculionidae | *Hylastes brunneus* | 38 |
| Curculionidae | *Hylastes cunicularius* | 138 |
| Curculionidae | *Hylastes opacus* | 10 |
| Curculionidae | *Hylesinus pilosus* | 92 |
| Curculionidae | *Hylesinus varius* | 70 |
| Curculionidae | *Hylobius abietis* | 43 |
| Curculionidae | *Hylobius excavatus* | 17 |
| Curculionidae | *Hylobius pinastri* | 32 |
| Curculionidae | *Hylurgops palliatus* | 53 |
| Curculionidae | *Ips typographus* | 7 |
| Curculionidae | *Magdalis carbonaria* | 8 |
| Curculionidae | *Phloeotribus spinulosus* | 51 |
| Curculionidae | *Pissodes gyllenhali* | 29 |
| Curculionidae | *Pissodes harcyniae* | 11 |
| Curculionidae | *Pissodes pini* | 11 |
| Curculionidae | *Pityogenes bidentatus* | 22 |
| Curculionidae | *Pityogenes chalcographus* | 97 |
| Curculionidae | *Pityogenes quadridens* | 10 |
| Curculionidae | *Pityophthorus micrographus* | 24 |
| Curculionidae | *Polygraphus poligraphus* | 24 |
| Curculionidae | *Polygraphus subopacus* | 12 |
| Curculionidae | *Tomicus piniperda* | 14 |
| Curculionidae | *Trypodendron domesticum* | 11 |
| Curculionidae | *Trypodendron lineatum* | 96 |
| Curculionidae | *Trypodendron signatum* | 73 |
| Dasytidae | *Aplocnemus tarsalis* | 12 |
| Elateridae | *Ampedus balteatus* | 19 |
| Elateridae | *Ampedus erythrogonus* | 62 |
| Elateridae | *Ampedus nigrinus* | 104 |
| Elateridae | *Denticollis linearis* | 45 |
| Elateridae | *Melanotus castanipes* | 123 |
| Erotylidae | *Dacne bipustulata* | 8 |
| Erotylidae | *Triplax russica* | 31 |
| Erotylidae | *Triplax scutellaris* | 5 |
| Eucnemidae | *Hylis procerulus* | 11 |
| Histeridae | *Plegaderus vulneratus* | 11 |
| Latridiidae | *Corticaria interstitialis* | 17 |
| Latridiidae | *Corticaria lapponica* | 19 |
| Latridiidae | *Corticaria longicornis* | 38 |
| Latridiidae | *Corticaria polypori* | 5 |
| Latridiidae | *Corticaria rubripes* | 56 |
| Latridiidae | *Enicmus fungicola* | 99 |
| Latridiidae | *Enicmus planipennis* | 60 |
| Latridiidae | *Latridius consimilis* | 35 |
| Latridiidae | *Latridius hirtus* | 10 |
| Latridiidae | *Stephostethus pandellei* | 41 |
| Latridiidae | *Stephostethus rugicollis* | 86 |
| Leiodidae | *Agathidium confusum* | 120 |
| Leiodidae | *Agathidium nigripenne* | 36 |
| Leiodidae | *Anisotoma axillaris* | 10 |
| Leiodidae | *Anisotoma castanea* | 69 |
| Leiodidae | *Anisotoma glabra* | 53 |
| Leiodidae | *Anisotoma orbicularis* | 6 |
| Leiodidae | *Leiodes oblonga* | 106 |
| Lucanidae | *Platycerus caprea* | 56 |
| Lycidae | *Dictyoptera aurora* | 101 |
| Lycidae | *Pyropterus nigroruber* | 13 |
| Lymexylidae | *Elateroides flabellicornis* | 17 |
| Lymexylidae | *Hylecoetus dermestoides* | 70 |
| Melandryidae | *Dolotarsus lividus* | 24 |
| Melandryidae | *Orchesia micans* | 16 |
| Melandryidae | *Orchesia minor* | 6 |
| Melandryidae | *Serropalpus barbatus* | 14 |
| Melandryidae | *Xylita laevigata* | 69 |
| Melyridae | *Dasytes niger* | 7 |
| Monotomidae | *Rhizophagus cribratus* | 14 |
| Monotomidae | *Rhizophagus depressus* | 9 |
| Monotomidae | *Rhizophagus dispar* | 59 |
| Monotomidae | *Rhizophagus ferrugineus* | 110 |
| Monotomidae | *Rhizophagus nitidulus* | 61 |
| Mycetophagidae | *Mycetophagus piceus* | 6 |
| Nitidulidae | *Cychramus luteus* | 40 |
| Nitidulidae | *Cychramus variegatus* | 16 |
| Nitidulidae | *Epuraea angustula* | 15 |
| Nitidulidae | *Epuraea biguttata* | 23 |
| Nitidulidae | *Epuraea binotata* | 32 |
| Nitidulidae | *Epuraea boreella* | 29 |
| Nitidulidae | *Epuraea contractula* | 7 |
| Nitidulidae | *Epuraea laeviuscula* | 7 |
| Nitidulidae | *Epuraea marseuli* | 82 |
| Nitidulidae | *Epuraea muehli* | 24 |
| Nitidulidae | *Epuraea oblonga* | 38 |
| Nitidulidae | *Epuraea pallescens* | 12 |
| Nitidulidae | *Epuraea pygmaea* | 113 |
| Nitidulidae | *Epuraea rufomarginata* | 43 |
| Nitidulidae | *Epuraea silacea* | 9 |
| Nitidulidae | *Epuraea terminalis* | 30 |
| Nitidulidae | *Epuraea thoracica* | 5 |
| Nitidulidae | *Epuraea variegata* | 17 |
| Nitidulidae | *Glischrochilus quadripunctatus* | 31 |
| Nitidulidae | *Ipidia binotata* | 13 |
| Nitidulidae | *Pityophagus ferrugineus* | 66 |
| Ptinidae | *Cacotemnus thomsoni* | 11 |
| Ptinidae | *Ptinus subpillosus* | 26 |
| Pyrochroidae | *Schizotus pectinicornis* | 6 |
| Salpingidae | *Rabocerus foveolatus* | 14 |
| Salpingidae | *Salpingus ruficollis* | 68 |
| Scarabaeidae | *Trichius fasciatus* | 7 |
| Scraptiidae | *Anaspis arctica* | 34 |
| Scraptiidae | *Anaspis bohemica* | 9 |
| Scraptiidae | *Anaspis frontalis* | 26 |
| Scraptiidae | *Anaspis marginicollis* | 111 |
| Scraptiidae | *Anaspis rufilabris* | 31 |
| Silvanidae | *Dendrophagus crenatus* | 27 |
| Silvanidae | *Silvanoprus fagi* | 51 |
| Sphaeritidae | *Sphaerites glabratus* | 18 |
| Sphindidae | *Aspidiphorus orbiculatus* | 68 |
| Sphindidae | *Sphindus dubius* | 8 |
| Staphylinidae | *Acrulia inflata* | 10 |
| Staphylinidae | *Actium californicum* | 8 |
| Staphylinidae | *Atheta lativentris* | 97 |
| Staphylinidae | *Atrecus pilicornis* | 42 |
| Staphylinidae | *Bibloporus bicolor* | 37 |
| Staphylinidae | *Bolitochara mulsanti* | 12 |
| Staphylinidae | *Dadobia immersa* | 39 |
| Staphylinidae | *Dinaraea aequata* | 6 |
| Staphylinidae | *Dropephylla linearis* | 13 |
| Staphylinidae | *Euplectus bescidicus* | 8 |
| Staphylinidae | *Euplectus karstenii* | 66 |
| Staphylinidae | *Euplectus mutator* | 7 |
| Staphylinidae | *Euplectus piceus* | 6 |
| Staphylinidae | *Euplectus punctatus* | 46 |
| Staphylinidae | *Euryusa castanoptera* | 9 |
| Staphylinidae | *Gabrius expectatus* | 25 |
| Staphylinidae | *Gyrophaena boleti* | 20 |
| Staphylinidae | *Gyrophaena fasciata* | 7 |
| Staphylinidae | *Holobus apicatus* | 9 |
| Staphylinidae | *Ischnoglossa elegantula* | 8 |
| Staphylinidae | *Leptusa pulchella* | 46 |
| Staphylinidae | *Lordithon speciosus* | 13 |
| Staphylinidae | *Microscydmus nanus* | 68 |
| Staphylinidae | *Oxyporus maxillosus* | 11 |
| Staphylinidae | *Phloeonomus pusillus* | 7 |
| Staphylinidae | *Phloeonomus sjobergi* | 18 |
| Staphylinidae | *Phloeopora corticalis* | 12 |
| Staphylinidae | *Phloeopora testacea* | 46 |
| Staphylinidae | *Phloeostiba lapponica* | 15 |
| Staphylinidae | *Placusa depressa* | 5 |
| Staphylinidae | *Placusa incompleta* | 6 |
| Staphylinidae | *Placusa tachyporoides* | 8 |
| Staphylinidae | *Platydracus affinis* | 19 |
| Staphylinidae | *Quedionuchus plagiatus* | 103 |
| Staphylinidae | *Quedius meng* | 21 |
| Staphylinidae | *Quedius xanthopus* | 134 |
| Staphylinidae | *Scaphisoma agaricinum* | 46 |
| Staphylinidae | *Scaphisoma subalpinum* | 7 |
| Staphylinidae | *Sepedophilus littoreus* | 80 |
| Staphylinidae | *Sepedophilus testaceus* | 13 |
| Staphylinidae | *Stenichnus bicolor* | 47 |
| Staphylinidae | *Xylostiba monilicornis* | 11 |
| Tenebrionidae | *Bolitophagus reticulatus* | 12 |
| Tenebrionidae | *Corticeus linearis* | 5 |
| Tetratomidae | *Hallomenus axillaris* | 5 |
| Tetratomidae | *Hallomenus binotatus* | 14 |
| Trogossitidae | *Peltis ferruginea* | 13 |
| Zopheridae | *Synchita humeralis* | 12 |

**Supporting references**

Burner, R.C., Stephan, J.G., Drag, L., Birkemoe, T., Muller, J., Snäll, T., Ovaskainen, O., Potterf, M., Siitonen, J., Skarpaas, O., Doerfler, I., Gossner, M.M., Schall, P., Weisser, W.W. & Sverdrup-Thygeson, A. (2021) Traits mediate environmental responses and species associations of forest beetles in ways that differ among bioclimatic regions. *Journal of Biogeography,* **48,** 3145-3157.

GBIF Secretariat (2021) GBIF Backbone Taxonomy. Checklist dataset. Accessed 2023-03-01 <https://doi.org/10.15468/39omei>.

Ovaskainen, O. & Abrego, N. (2020) *Joint Species Distribution Modelling: With Applications in R*. Cambridge University Press, Cambridge.
